# Temporal Changes in Alzheimer's Disease‐Related Biomarkers in the CSF of Cognitively Normal Subjects at Different Ages: The Chongqing Ageing and Dementia Study

**DOI:** 10.1111/acel.70036

**Published:** 2025-03-09

**Authors:** Wei‐Wei Li, Dong‐Yu Fan, Qi Sun, Lei‐Kai Wang, Bing‐Qiang Huang, Zhong‐Yuan Yu, Ding‐Yuan Tian, Ying‐Ying Shen, Cheng‐Rong Tan, Gui‐Hua Zeng, Fan Zeng, Jin Fan, Zhen Wang, Yan‐Jiang Wang, Jun Wang

**Affiliations:** ^1^ Department of Neurology and Centre for Clinical Neuroscience, Daping Hospital Third Military Medical University Chongqing China; ^2^ Department of Neurology The General Hospital of Western Theater Command Chengdu China; ^3^ Department of Plateau Diseases, Shigatse Branch, Xinqiao Hospital Third Military Medical University Shigatse China; ^4^ Department of Anesthesiology, Daping Hospital Third Military Medical University Chongqing China; ^5^ Department of Neurosurgery The General Hospital of Western Theater Command Chengdu China; ^6^ Chongqing Key Laboratory of Aging and Brain Diseases Chongqing China

**Keywords:** aging, Alzheimer's disease, *APOE* ε4, Aβ, biomarkers, cerebrospinal fluid, tau, trajectory

## Abstract

Revealing the temporal evolution of cerebrospinal fluid (CSF) biomarkers during aging is critical to understanding disease pathogenesis and developing early diagnoses and interventions for Alzheimer's disease (AD). CSF was obtained from 549 cognitively normal subjects between 18 and 93 years of age. 12 AD‐related biomarkers were evaluated, including amyloid β (Aβ42, Aβ40, Aβ42/Aβ40 ratio), hyperphosphorylated tau (P‐tau), neuronal injury/degeneration (T‐tau, NFL, NSE, H‐FABP, VILIP‐1), neuroinflammation biomarkers (YKL‐40, TREM2), and α‐synuclein (α‐synuclein). Associations between these biomarkers and age as well as apolipoprotein E (*APOE*) ε4 status were evaluated, and the associations among biomarkers were assessed. CSF Aβ42, P‐tau, and T‐tau levels exhibited nonlinear associations with age, among which Aβ42 was significantly modulated by *APOE* ε4 status. Specifically, an accelerated decline in Aβ42 levels occurred at 45.69 years of age in the *APOE* ε4+ group, which was almost 23 years earlier than that in the *APOE* ε4− group (68.02 years). The age‐related change pattern of CSF P‐tau is similar to that of T‐tau, with both increasing slightly with age but showing an accelerated change at ≈60 years of age in the *APOE* ε4+ group. All the other biomarkers except for α‐synuclein were linearly associated with age, and *APOE* ε4 status had no effect on these associations. Most biomarkers were positively correlated with each other except for Aβ42/Aβ40 ratio. The evolution of AD‐related biomarkers in CSF varies throughout the adult lifespan, with the APOE ε4 allele modifying the temporal changes in CSF Aβ42 levels, as well as potentially influencing P‐tau and T‐tau levels.

## Graphical abstract

The APOE ε4 allele modifies the temporal change in CSF Aβ42 levels among cognitively normal individuals, with the APOE ε4+ group showing an accelerated decline almost 20 years earlier than the APOE ε4− group.

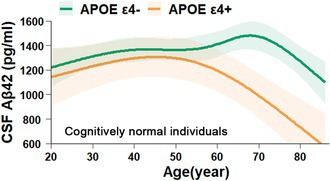

## 1 Introduction

Alzheimer's disease (AD) is the most common type of aging‐related dementia and imposes a heavy burden on patients and society (Mukadam et al. 2024). AD is irreversible once patients have entered the dementia stage, and early diagnosis and intervention are critical for preventing, slowing, or halting disease progression. Neuropathologic abnormalities in biomarker levels can begin 15–20 years before the clinical manifestations of AD (Jia et al. 2024). Understanding the temporal evolution of AD pathology during the aging process can help define when the disease starts, thus providing an appropriate time point for early intervention (Frisoni et al. 2024; Jia et al. 2023).

A pathological hallmark of AD is the presence of an extracellular senile plaque in the brain, which is primarily composed of amyloid‐beta (Aβ) (Blennow et al. 2006). Decreases in the Aβ42 or Aβ42/Aβ40 ratio in cerebrospinal fluid (CSF) are critical biomarkers that reflect Aβ deposition in the brain (Jack Jr. et al. 2018), highlighting the importance of detecting the earliest change in CSF Aβ42 or the Aβ42/Aβ40 ratio for the early diagnosis of AD. In addition, AD is associated with other brain downstream pathologies (Baiardi et al. 2024; Hampel et al. 2021; Selkoe and Hardy 2016; Ulrich et al. 2017; Wisse et al. 2024), such as intraneuronal neurofibrillary tangles, neuronal injury/degeneration, neuroinflammation, and copathologies. Thus, investigations into the temporal evolution of biomarkers reflecting the above pathologies (Chiasserini et al. 2017; Filipello et al. 2022; Gaur et al. 2023; Khalil et al. 2024; Mahaman et al. 2022; Mavroudis, Chowdhury, et al. 2021; Mavroudis, Petridis, et al. 2021; Ossenkoppele et al. 2022; Schmidt et al. 2014) are essential for understanding AD pathogenesis and for early diagnosis.

Aging is an important risk factor for AD. Heritability accounts for a large proportion of all AD cases (Bellenguez et al. 2022; Reitz et al. 2023), and the apolipoprotein E (*APOE*) ε4 allele is the major susceptibility gene for sporadic AD (sAD) (Reiman et al. 2020). Elucidating how AD‐related biomarkers evolve during aging and how the *APOE* genotype affects this process is helpful for understanding disease pathogenesis and early diagnosis.

In this study, we aimed to examine the temporal changes in AD‐related biomarkers in CSF and whether *APOE* ε4 modifies their temporal evolution in a cognitively normal (CN) cohort across different ages. Specifically, we aimed to: Characterize the temporal progression and sequence of AD biomarker trajectories across the adult lifespan; identify critical inflection points in the acceleration of biomarker changes to delineate the sequence of pathological events; elucidate whether the *APOE* ε4 allele modifies the temporal dynamics of biomarkers.

## 2 Methods

### 2.1 Study Population

Consecutive subjects with normal cognition admitted to Daping Hospital between January 2018 and October 2018 were enrolled in the present study. The participants who showed no evidence of cognitive impairment were classified as CN. Eligible subjects included those who (1) would accept lumbar anesthesia for surgery owing to diseases of the urinary system or who were willing to undergo lumbar puncture; (2) were aged ≥ 18 years; (3) had no medical history of stroke or other neurological diseases; and (4) were willing to participate in the study. A total of 549 subjects met the enrollment standards and were included in our study. *APOE* genotyping was performed for 519 subjects (30 missing), and *APOE* ɛ4 status was categorized as positive (presence of ɛ4 alleles) or negative (absence of ɛ4 alleles). The following variables were collected as potential effect modifiers (defined as covariates): sex, education level, *APOE* ε4 status (*APOE* ε4− vs. *APOE* ε4+), smoking status, alcohol status, hyperlipidemia, hypertension, diabetes, and coronary heart disease (CHD). This study was approved by the Institutional Review Board of Daping Hospital, and all subjects and their caregivers provided informed consent.

### 2.2 CSF Sampling and Analyses

Our laboratory is a centre of the Alzheimer's Association quality control programme for CSF and blood biomarkers (Mattsson et al. 2011), and the collection, storage, and examination of CSF biomarkers were conducted in accordance with standardized procedures (Blennow et al. 2006). Specifically, the CSF samples were collected in low‐binding polypropylene tubes, centrifuged at 1800 × *g* at room temperature for 10 min. Within 2 h after collection, the samples were aliquoted and stored frozen at −80°C until analysis. All the samples were free from blood contamination.

Commercially available enzyme‐linked immunosorbent assay (ELISA) kits were used to test the levels of CSF Aβ42, Aβ40, P‐tau181, and T‐tau (Fujirebio‐Europe, Gent, Belgium), and these factors have previously been validated in multiple studies and have shown reliable trial sensitivity and intra‐ and interassay precision (Jansen et al. 2015). The NFL levels were measured with an ultrasensitive single‐molecule array (SIMOA) on a Simoa HD‐1 analyzer (Quanterix, Lexington, Massachusetts). Other biomarker analyses were also performed with the following ELISA kits: NSE (R&D Systems, USA), H‐FABP (Hycult Biotech, Netherlands), VILIP‐1 (BioVendor, Czech Republic), YKL‐40 (Raybiotech, USA), TREM2 (Qarigo, China), and α‐synuclein (Biolegend, USA). The detailed information for these ELISA kits was provided in Table S1. All experiments were performed according to the manufacturer's protocol by experienced laboratory technicians who were completely blinded to the clinical information. The inter‐assay coefficients of variation (CV) for all biomarkers were within the accepted range (0.71% ~ 15.07%). Owing to the limited sample volume, each sample was assessed using a single measurement. All sample levels were within the range of the standards, except for VILIP‐1. For VILIP‐1, samples with concentrations below the detection limit were assigned a value equal to the lower limit of detection (27 pg/mL) of the BioVendor ELISA kit. This approach ensured data completeness while accounting for the assay's detection threshold.

### 2.3 Statistical Analysis

Normality of distribution was assessed using both visual inspection of the Q‐Q plot and the Shapiro–Wilk test. Differences between groups were evaluated with chi‐square tests for categorical variables and independent samples *t* tests or Mann–Whitney *U* tests for continuous variables according to the distribution characteristics of the data (Table 1). For each biomarker model, participants were excluded if they had one or more missing data points at the biomarker level. To address missing data in covariates including *APOE* status, smoking status, alcohol status, hyperlipidemia, hypertension, diabetes, and CHD, we employed multiple imputation using the Multiple Imputation by Chained Equations (MICE) algorithm in SPSS (Costantini et al. 2023). Five imputed datasets were generated and analyzed individually. The model with the smallest Akaike Information Criterion (AIC) value was selected for the final analysis. Results from the other datasets were consistent with those from the selected model. Education data were unavailable for 246 participants; therefore, the education level variable was omitted from the adjustment analyses. The distribution of missing data is detailed in Table S2.

**TABLE 1:** Demographics and biochemical data according to *APOE* ε4 status.

Characteristics	Total (*n* = 549)	*APOE* ε4+ group (*n* = 104)	*APOE* ε4− group (*n* = 415)	*p*
Age, mean (range), year	54.34 (18–93)	55.23 (18–93)	55.25 (18–89)	0.87
Sex, M/F (% male)	426/123 (77.6)	84/20 (80.8)	324/91 (78.1)	0.60
Smoke, *n* (%)	213 (38.8)	44 (42.3)	162 (39.0)	0.58
Alcohol, *n* (%)	112 (20.4)	23 (22.1)	86 (20.7)	0.79
Hyperlipemia, *n* (%)	5 (0.9)	20 (19.2)	88 (21.2)	1.00
Hypertension, *n* (%)	110 (20)	1 (1.0)	4 (1.0)	0.69
Diabetes, *n* (%)	44 (8.0)	7 (6.7)	37 (8.9)	0.56
CHD, *n* (%)	28 (5.1)	9 (8.7)	19 (4.6)	0.14
Aβ42, pg/mL, mean (SD)	1331.28 (402.98)	1218.81 (429.56)	1363.89 (393.80)	**0.002**
Aβ40, pg/mL, mean (SD)	10722.06 (4355.76)	10701.78 (4327.11)	10789.84 (4367.99)	0.84
Aβ42/Aβ40 ratio, mean (SD)	0.14 (0.09)	0.13 (0.08)	0.15 (0.10)	**0.049**
P‐tau, pg/mL, mean (SD)	42.03 (16.81)	43.29 (16.03)	41.94 (16.92)	0.19
T‐tau, pg/mL, mean (SD)	179.76 (103.94)	193.70 (100.27)	177.86 (103.85)	0.06
NFL, pg/mL, mean (SD)	1386.95 (1373.35)	1315.21 (1084.28)	1381.63 (1359.60)	0.46
NSE, pg/mL, mean (SD)	7724.85 (3265.12)	8173.31 (3309.70)	7617.66 (3228.63)	0.16
H‐FABP, pg/mL, mean (SD)	314.93 (172.51)	327.24 (147.19)	314.98 (182.14)	0.09
VILIP‐1, pg/mL, mean (SD)	128.76 (72.39)	140.94 (79.20)	126.35 (68.85)	0.18
YKL‐40, ng/mL, mean (SD)	393.77 (207.68)	378.29 (194.56)	396.93 (209.14)	0.45
TREM2, pg/mL, mean (SD)	9779.88 (4911.46)	8799.56 (4258.31)	9924.58 (4957.56)	**0.04**
α‐synuclein, pg/mL, mean (SD)	618.32 (498.35)	511.35 (413.05)	644.09 (523.24)	**0.03**

*Note:* Data differences were evaluated via chi‐squared tests for categorical variables and independent samples *t* tests or Mann–Whitney *U* tests for continuous variables according to the distribution characteristics of the data. The bolded *p*‐value indicates significant result.

Abbreviations: *APOE*, apolipoprotein E; CHD, coronary heart disease history; F, female; M, male; SD, standard deviation.

Linear regression analyses and restricted cubic splines (RCSs) using knots based on the AIC were simultaneously performed to model the associations between biomarkers and age, and the optimum values were displayed with fitted lines or curves. For the RCS, the knot locations were pre‐specified on the basis of the distribution of ages in our study and served as the reference points to support the majority of flexible nonlinear curves, following the strategies of Harrell (Boyd 2005). The maximum likelihood estimator to the inflection point was obtained, along with its 95% confidence interval (CI). The inflection point represents a turning point or boundary for the association between the dependent and independent variables. The inflection point was defined as the age at which the dependent variable appeared to start diverging. Specifically, visual inspection provides a preliminary assessment of the curve's shape (e.g., U‐shaped, inverted U‐shaped) (Harrell 2012; Jia et al. 2024). The rcssci package in R helps precisely locate the inflection points by solving the second derivative of the spline function. For linear regression analyses, we constructed three linear regression models: the first without adjustment for covariates, the second adjusted for sex and *APOE* ε4 status, and the third adjusted for all the covariates aforementioned. Standardized coefficients (β) and *p* values were calculated for the models. Of note, biomarkers were log‐transformed when necessary to meet the “LINE” standards of residual including linearity, independence, normality, and homoscedasticity. However, untransformed data were presented in figures to aid in the interpretation of biomarker trajectories.

To assess whether *APOE* ɛ4 status affects the associations between age and AD biomarkers, we conducted two analyses. Firstly, we included an interaction term between *APOE* ε4 status (code as 1 for carriers and 0 for non‐carriers) and age in the RCS and linear regression models (rms package for RCS, reghelper and sjPlot package for linear regression). Secondly, we independently repeated the similar analyses described above by stratifying the *APOE* genotype (*APOE* ɛ4+ vs. *APOE* ɛ4−). All analyses were performed adjusting for all the aforementioned covariates. Sex‐specific subgroup analyses were also performed to explore the age‐related changes of biomarkers by sex.

Spearman correlation analyses were conducted to examine the correlations among the various biomarkers, and a heatmap was used to visually display these relationships. To examine whether *APOE* ε4 status modifies cross‐sectional associations between CSF Aβ42 and P‐tau, T‐tau, NFL, NSE, H‐FABP, VILIP‐1, YKL‐40, TREM2, and α‐synuclein, we used covariate‐adjusted linear regression models to test for interactions between *APOE* ε4 status (*APOE* ε4+ vs. *APOE* ε4−) and Aβ42 with respect to each of the aforementioned biomarkers. Notably, age was also included in the covariates to covary its influence. Given the predictable different age‐related change patterns of Aβ42 before and after the inflection point in RCS analysis, we performed exploratory analyses of the effect of age on the associations between Aβ42 and the aforementioned biomarkers, using models treating age as a dichotomous variable and using the inflection time point of Aβ42 found for the total cohort.

All hypothesis testing was two‐sided, and a *p* value less than 0.05 was considered statistically significant. All the statistical computations and figures were performed via SPSS version 26.0 (SPSS Inc., IBM) and R software (version 4.4.0, R Foundation for Statistical Computing, Vienna, Austria).

## 3 Results

### 3.1 Demographic and Biochemical Data of the Study Subjects

The demographic characteristics and CSF biomarker data are shown in Table 1. Among the 549 participants, 22.4% were female; the mean age was 54.34 years, ranging from 18 to 93 years. Among the subjects whose *APOE* information was available, 20.0% were *APOE* ε4 carriers. About 0.9% of the subjects have hyperlipemia, 8.0% have diabetes, and 5.1% have CHD. Many participants reported smoking history (38.8%). Sex, smoking status, alcohol status, hypertension, diabetes, hyperlipidemia, and CHD were similarly distributed in each subgroup (*p* > 0.05). Among all the biomarkers we tested, Aβ42 (*APOE* ε4+ vs. *APOE* ε4−: 1218.81 pg/mL vs. 1363.89 pg/mL, *p =* 0.002), the Aβ42/Aβ40 ratio (*APOE* ε4+ vs. *APOE* ε4−: 0.13 vs. 0.15, *p =* 0.049), TREM2 (*APOE* ε4+ vs. *APOE* ε4−: 8799.56 vs. 9924.58, *p =* 0.04) and α‐synuclein (*APOE* ε4+ vs. *APOE* ε4−: 511.35 vs. 644.09, *p =* 0.03) significantly differed between the *APOE* ε4 subgroups.

### 3.2 Associations Between Biomarkers and Age in the Total Cohort

**FIGURE 1:**
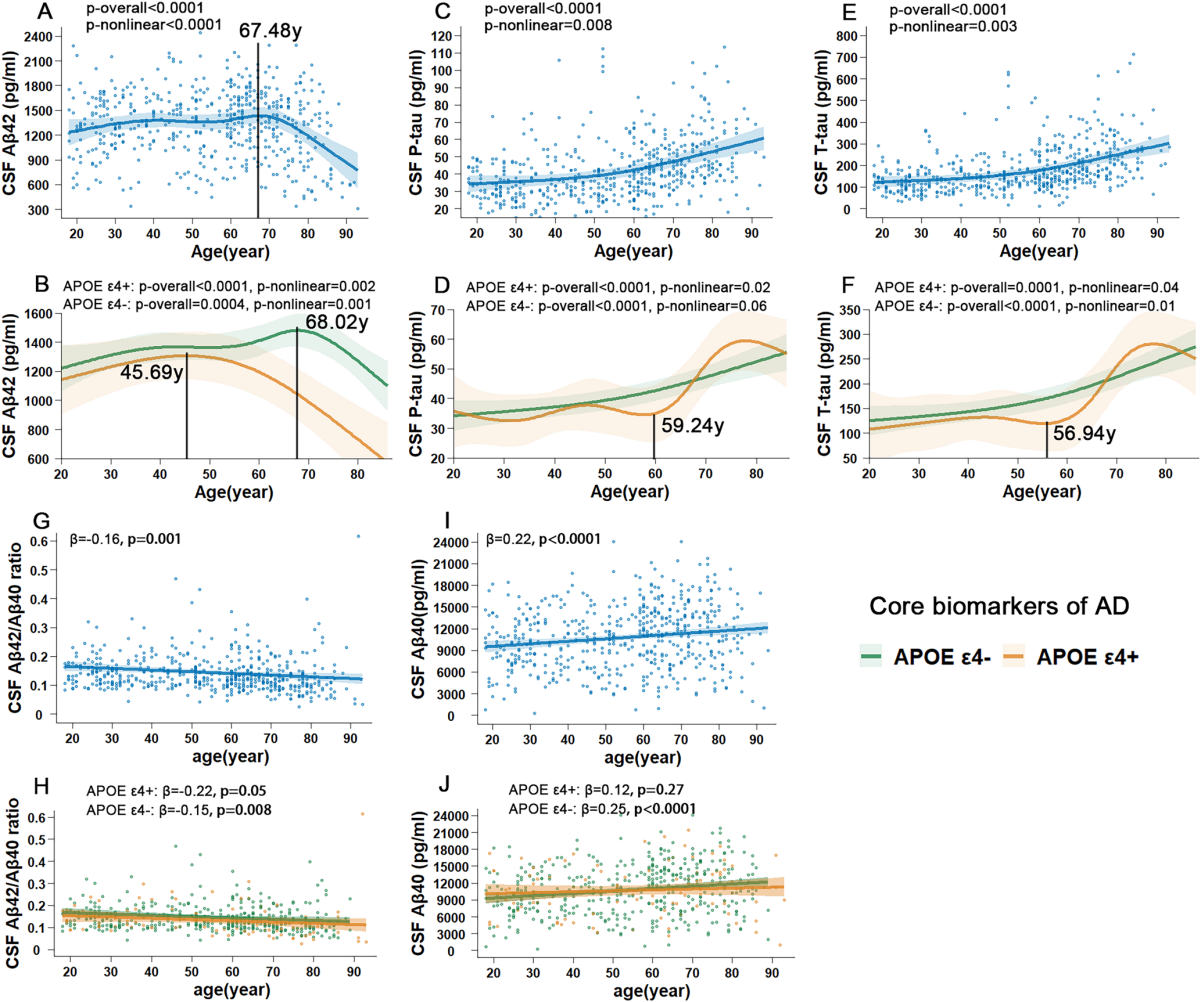
*APOE* differences in the associations between core biomarkers and age. Total and *APOE* ɛ4‐stratified analyses of age‐related changes in CSF Aβ42 (A, B), P‐tau (C, D), and T‐tau (E, F) were modeled with RCSs adjusted by covariates. Here, *p*‐overall < 0.05 referred to a significant association (linear or nonlinear), and *p*‐nonlinear < 0.05 referred to a nonlinear association between biomarkers and age. The inflection points of age are labeled with black vertical lines. Aβ42/Aβ40 ratio (G, H) and Aβ40 (I, J) were modeled with linear regressions adjusted by covariates. Shading around the curve or regression line represents standard errors. β, standardized coefficient; *APOE*, apolipoprotein E; RCS, restricted cubic spline.

**FIGURE 2:**
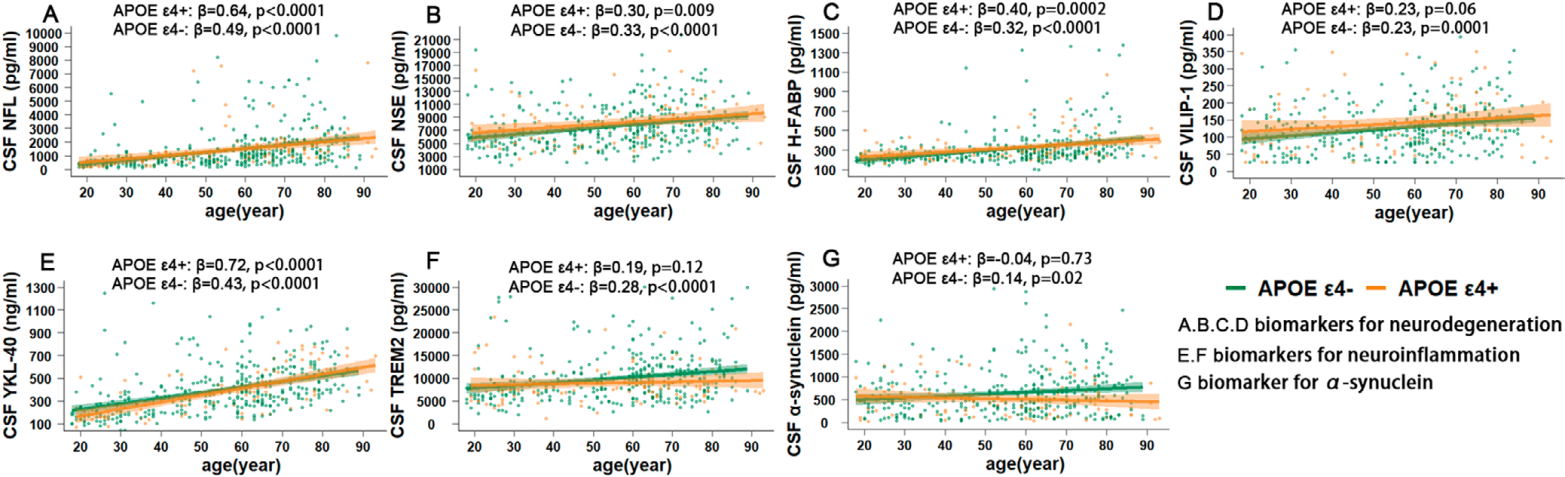
*APOE* differences in the linear associations between other biomarkers and age. *APOE* ε4‐based stratified analyses of the age‐related changes in the CSF NFL (A), NSE (B), H‐FABP (C), VILIP‐1 (D), YKL‐40 (E), TREM2 (F) and α‐synuclein (G) were modeled with linear regressions adjusted by covariates. Shading around the regression line represents standard errors. β, standardized coefficient; APOE, apolipoprotein E.

RCS revealed nonlinear associations (defined as *p*‐nonlinear < 0.05) between Aβ42 (*p*‐nonlinear < 0.0001), P‐tau (*p*‐nonlinear = 0.008), and T‐tau (*p‐*nonlinear = 0.003) and age (Figure 1, Table S3). We identified a prominent inflection point for Aβ42 (at the age of 67.48 years, Figure 1A). Specifically, an accelerated decline in Aβ42 occurred at that time point, representing a turning point in the relationship between Aβ42 and age. Both P‐tau (Figure 1C) and T‐tau (Figure 1E) levels exhibited gradually increasing temporal evolution, with no prominent visual turning points. For the other biomarkers (Aβ40, Aβ42/Aβ40 ratio, NFL, NSE, H‐FABP, VILIP‐1, YKL‐40, TREM2 and α‐synuclein) that lacked significance in the RCS analyses (*p*‐nonlinear > 0.05, Table S3), Three linear regressions were performed as described above, and the results across the models were consistent (Table 2). All biomarkers except for α‐synuclein demonstrated significant linear associations with age, with Aβ42/Aβ40 ratio showing a descending pattern and others showing growth patterns with age (Figures 1 and 2). Segmented linear regressions divided by the inflection point of 67.48 years were also performed for Aβ42 levels owing to the opposite correlations on either side of the time point (Table 2). Taking Model 3 as an example, Aβ42 levels increased among individuals ≤ 67 years old (*p =* 0.02, β = 0.14) and decreased among individuals > 67 years old (*p <* 0.0001, β = −0.30).

**TABLE 2:** Multiple linear regression models for the associations between biomarkers and age.

CSF biomarkers	Log‐transformed	Model 1		Model 2		Model 3	
		β	*p*	β	*p*	β	*p*
Aβ42 (age ≤ 67 years)	No	0.13	**0.01**	0.13	**0.02**	0.14	**0.02**
Aβ42 (age > 67 years)	No	−0.34	**< 0.0001**	−0.31	**< 0.0001**	−0.3	**< 0.0001**
Aβ40	No	0.16	**< 0.0001**	0.16	**< 0.0001**	0.22	**< 0.0001**
Aβ42/Aβ40 ratio	No	−0.12	**0.005**	−0.12	**0.006**	−0.16	**0.001**
P‐tau	Yes	0.39	**< 0.0001**	0.39	**< 0.0001**	0.4	**< 0.0001**
T‐tau	Yes	0.43	**< 0.0001**	0.43	**< 0.0001**	0.44	**< 0.0001**
NFL	Yes	0.54	**< 0.0001**	0.54	**< 0.0001**	0.52	**< 0.0001**
NSE	No	0.28	**< 0.0001**	0.27	**< 0.0001**	0.32	**< 0.0001**
H‐FABP	No	0.33	**< 0.0001**	0.33	**< 0.0001**	0.33	**< 0.0001**
VILIP‐1	No	0.22	**< 0.0001**	0.22	**< 0.0001**	0.22	**< 0.0001**
YKL‐40	No	0.48	**< 0.0001**	0.48	**< 0.0001**	0.49	**< 0.0001**
TREM2	Yes	0.25	**< 0.0001**	0.25	**< 0.0001**	0.25	**< 0.0001**
α‐synuclein	Yes	0.08	0.11	0.07	0.16	0.09	0.08

*Note:* Model 1: no covariates adjusted; Model 2: adjusted for sex, *APOE* ε4 status (*APOE* ε4− vs. *APOE* ε4+); Model 3: adjusted for sex, *APOE* ε4 status (*APOE* ε4− vs. *APOE* ε4+), smoking status, alcohol status, hyperlipemia, hypertension, diabetes, and CHD. Segmented linear regressions divided by the inflection point of 67.48 years were performed for Aβ42 given the opposite correlations on either side of the inflection point. The bolded *p*‐value indicates significant result.

Abbreviations: β, standardized coefficient; CSF, cerebrospinal fluid; y, years of age.

### 3.3 Impact of *APOE* Genotypes on the Associations Between Biomarkers and Age

In the total cohort (Tables S3, S4), we observed significant interaction effects between *APOE* ε4 status and age on CSF Aβ42 (*p* for interaction = 0.003) and α‐synuclein (*p* for interaction = 0.05, borderline statistical significance), indicating that the *APOE* ε4 status modified the temporal evolution of the two biomarkers. No interaction effects were found for other biomarkers. Interaction analyses of sex and age indicated that the changes in NFL and NSE with age might potentially differ between sexes (Table S7, Figures S1 and S2).

In the *APOE*‐stratified analysis of Aβ42 (Figure 1B), the inflection point was observed at the age of 45.69 years in the *APOE* ε4+ group (*p*‐nonlinear = 0.002) and at the age of 68.02 years in the *APOE* ε4− group (*p*‐nonlinear = 0.001), indicating that the Aβ42 levels of *APOE* ε4+ subjects may have started an accelerated decline almost 23 years earlier than those noted in *APOE* ε4− subjects. Segmented linear regressions divided by the inflection points were then performed within each *APOE* ε4 subgroup to quantify the correlations. In the *APOE* ε4+ subgroup, Aβ42 initially showed a nonsignificant increasing trend with age (*n* = 43, *p =* 0.25, β = 0.21) before the age of 45.69 years and then decreased rapidly (*n* = 70, *p <* 0.0001, β = −0.57). In the *APOE* ε4− subgroup, Aβ42 initially gradually increased with age (*n* = 317, *p =* 0.005, β = 0.17) before the age of 68.02 years but decreased after that time point (*n* = 119, *p =* 0.006, β = −0.26), with a slower descent speed than that noted for the *APOE* ε4+ subgroup.

The age‐related change in P‐tau levels (Figure 1D) is similar to that of T‐tau levels (Figure 1F) in both the *APOE* ε4+ and *APOE* ε4− groups. In the *APOE* ε4− group, P‐tau (*p*‐nonlinear = 0.06, borderline statistical significance) and T‐tau (*p‐*nonlinear = 0.01) gradually increased, with no prominent visual turning points. In the *APOE* ε4+ group, inflection points were identified at age 59.24 years for P‐tau (*p‐*nonlinear = 0.02) and at age 56.94 years for T‐tau (*p‐*nonlinear = 0.04), suggesting an almost simultaneous acceleration of change at the approximate age of 60 years.

In *APOE*‐stratified analyses of other biomarkers that displayed linear correlations (Figures 1 and 2, Table S5), CSF Aβ40 (*p =* 0.27, β = 0.12), VILIP‐1 (*p =* 0.06, β = 0.23), TREM2 (*p =* 0.12, β = 0.19) and α‐synuclein (*p =* 0.73, β = −0.04) exhibited no associations with age in the *APOE* ε4+ group, whereas all the other biomarkers exhibited significant correlations with age in both the *APOE* ε4+ and *APOE* ε4− subgroups (*p <* 0.05; β < 0 for the Aβ42/Aβ40 ratio and β > 0 for other biomarkers).

### 3.4 Associations of Biomarkers With Each Other

**FIGURE 3:**
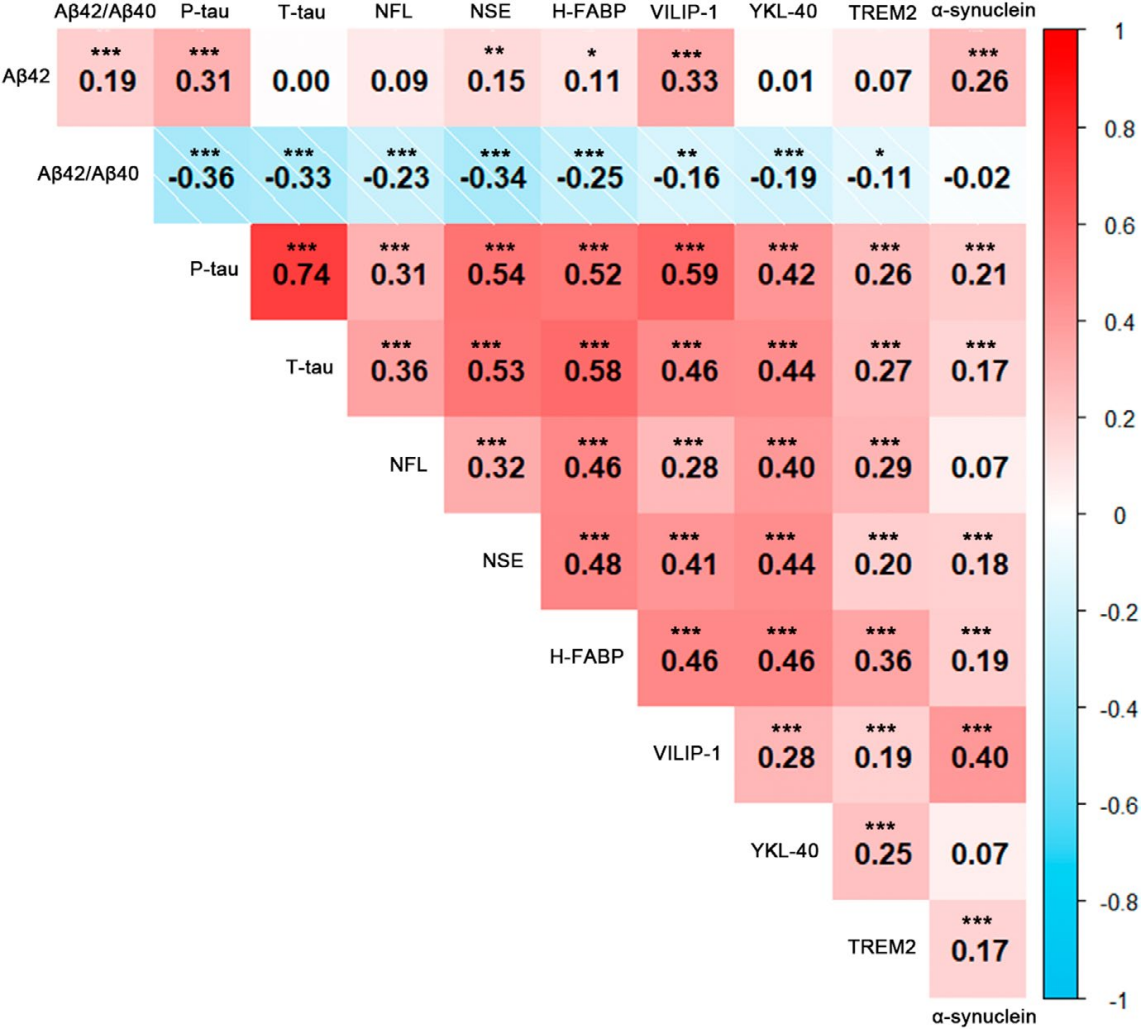
Associations among various biomarkers. Spearman correlation analyses across each pairing of biomarkers are displayed with a correlation heatmap. The numbers within each square represent the correlation coefficients (*r*). The asterisks on top of the correlation coefficients mean significant correlations (*p < 0.05, **p < 0.01; ***p < 0.001). Red square coloring means positive correlation; blue square coloring means negative correlation.

We performed spearman correlation analyses across each pairing of biomarkers and displayed the results with correlation heatmap (Figure 3) and correlation matrix table (Table S6). As expected, we saw significant negative correlations between Aβ42/Aβ40 ratio and other biomarkers with the exception of α‐synuclein. Apart from the following pairings, which showed no significant correlation: Aβ42 vs. T‐tau, Aβ42 vs. NFL, Aβ42 vs. YKL‐40, Aβ42 vs. TREM2, NFL vs. α‐synuclein and YKL40 vs. α‐synuclein, all other variable pairings were positively correlated with each other. Notable, P‐tau and T‐tau were positively associated with a strong association (*p <* 0.001; β = 0.74).

### 3.5 Associations Between Aβ42 and Other Biomarkers in Stratified Age Groups

Given that Aβ42 levels showed different patterns of change before and after the inflection point (67.48 years), the data were further stratified into two groups: a younger group (age ≤ 67 years, *n* = 387) and an older group (age > 67 years, *n* = 162). Linear regressions adjusted for age as well as covariates were performed to assess the associations between Aβ42 and the other biomarkers separately in the younger and older groups to reduce the impact of age and to explore the relationships within different age ranges. We first examined *APOE* differences in the associations between Aβ42 and the other biomarkers and found no interaction effects on any of the age‐stratified subgroups (*p* for interaction > 0.05). Therefore, *APOE* ε4 status was used as a covariate instead of being used for the construction of *APOE* ε4‐based stratified analyses.

**FIGURE 4:**
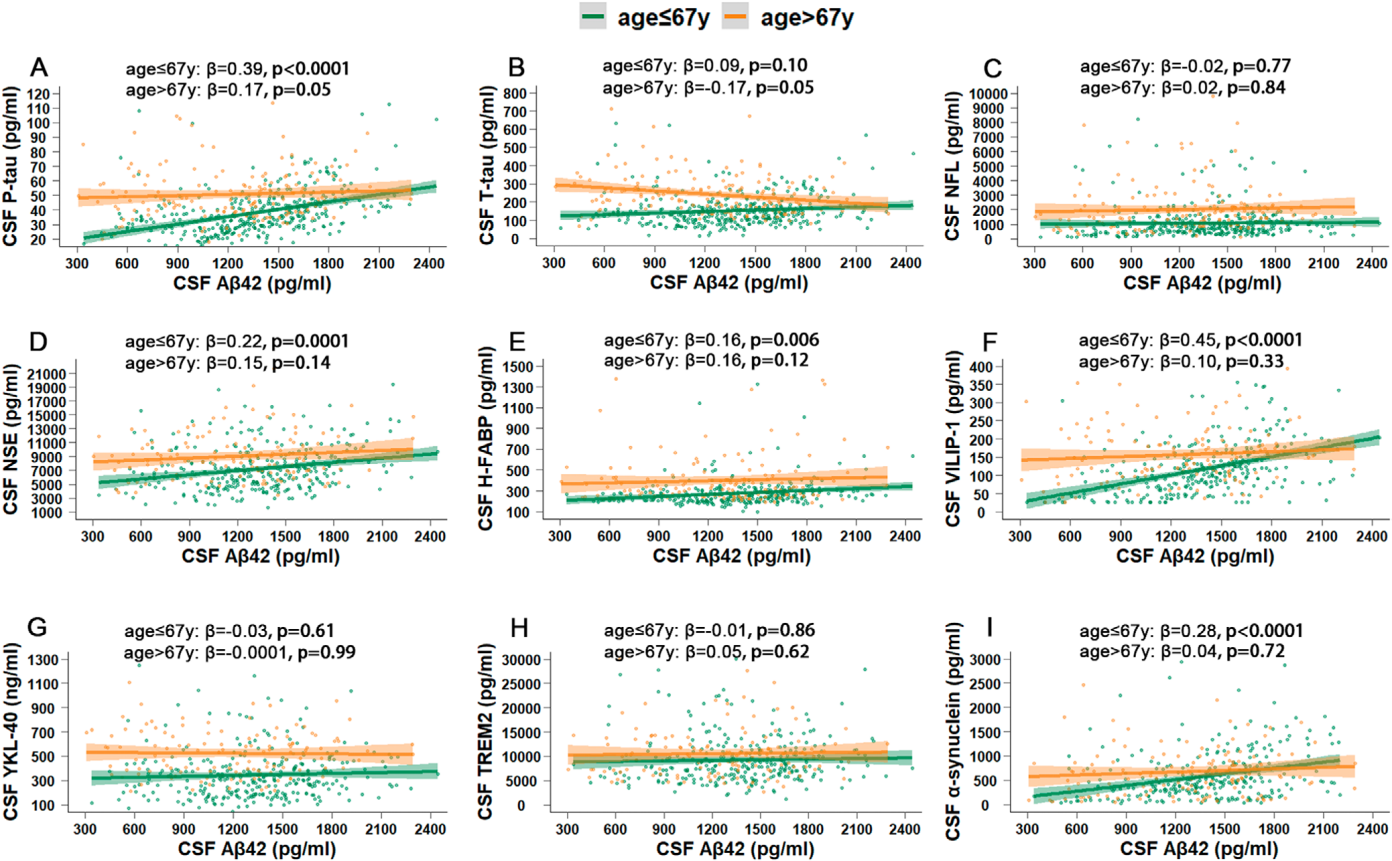
Associations between Aβ42 and other biomarkers in stratified age groups. The correlations between CSF Aβ42 and P‐tau (A), T‐tau (B), NFL (C), NSE (D), H‐FABP (E), VILIP‐1 (F), YKL‐40 (G), TREM2 (H), and α‐synuclein (I) were displayed with linear regressions adjusted by covariates. All analyses were performed in stratified age groups according to the inflection point of Aβ42 in the RCS curve (age ≤ 67 vs. age > 67 years). Shading around the regression line represents standard errors. β, Standardized coefficient; y, year.

As shown in Figure 4, in the younger group, P‐tau (*p <* 0.0001, β = 0.39), NSE (*p <* 0.0001, β = 0.22), H‐FABP (*p =* 0.006, β = 0.16), VILIP‐1 (*p <* 0.0001, β = 0.45) and α‐synuclein (*p <* 0.0001, β = 0.28) levels were positively associated with Aβ42 levels, whereas T‐tau, NFL, YKL‐40, and TREM2 levels were not associated with Aβ42 levels. In the older group, only P‐tau (*p =* 0.05, β = 0.17) and T‐tau (*p =* 0.05, β = −0.17) levels were marginally associated with Aβ42 levels. Significant interactions were found between age (age ≤ 67 vs. age > 67 years) and Aβ42 levels on P‐tau (*p* for interaction = 0.0007), T‐tau (*p* for interaction = 0.001), VILIP‐1 (*p* for interaction = 0.0006) and α‐synuclein (*p* for interaction = 0.03) levels, indicating significant differences between stratified age groups. No interaction effects were observed between age and Aβ42 levels on the other biomarkers, suggesting similar associations within different age ranges.

## 4 Discussion

In this cross‐sectional study, we investigated the age‐related changes in multiple AD‐related biomarkers in CSF and the associations between Aβ42 and other biomarkers. As reflected by our findings, aging is correlated with all the biomarkers we studied, supporting the effects of aging on these AD‐related pathologies, including amyloid (Aβ42, Aβ40, Aβ42/Aβ40 ratio), tangle pathology (P‐tau), neuronal injury/degeneration (T‐tau, NFL, NSE, VILIP and H‐FABP), neuroinflammation (YKL‐40, TREM2) and α‐synuclein (α‐synuclein). Chronic inflammation is a process that spans the entire lifespan and promotes neurodegenerative disorders (Furman et al. 2019). Our findings that YKL‐40 and TREM2, both markers of neuroinflammation, increased with age support the concept. Additionally, our study highlights the role of aging in the metabolism of α‐synuclein, which is a biomarker of Parkinson's disease (PD). It suggests that aging and chronic inflammation may all interact to drive the AD process. Our study underscores the necessity for a comprehensive approach to understanding these interactions, particularly in the context of aging.

Our study revealed a nonlinear association of CSF Aβ42 levels with age, which was modulated by *APOE* ε4 status. Aβ42 levels decreased after the age of 45.69 years in *APOE* ε4+ carriers, almost 23 years earlier than they did in the *APOE* ε4− group, suggesting a considerable impact of *APOE* ε4 on Aβ pathology metabolism (Saddiki et al. 2020). In contrast to other biomarkers, decreased Aβ42 levels and a decreased Aβ42/Aβ40 ratio in CSF indicate increased Aβ plaque deposition in the brain, and the age at which CSF Aβ42 levels begin to decrease might be the starting point for brain amyloid deposition in the preclinical stage of AD (Morris et al. 2010). Therefore, as our study suggested, the deposition of Aβ plaques in *APOE* ε4 carriers occurs far earlier than originally assumed, likely starting at middle age, which is consistent with the findings of a previous study (Luo et al. 2022), supporting the pathogenic role of *APOE* ε4 in the pathogenesis of AD (Fortea et al. 2024). In addition, compared with *APOE* ε4− individuals, *APOE* ε4 carriers had a faster rate of reduction after the inflection point. Our findings, along with those of previous studies (Fortea et al. 2024; Insel et al. 2021; Lautner et al. 2017; Li et al. 2017; Luo et al. 2022; Resnick et al. 2015; Saddiki et al. 2020), emphasize the importance of *APOE* ε4 status on age‐related changes in Aβ42. However, these conclusions exhibit considerable discrepancies, probably influenced by the subject inclusion standard, ethnicity, biomarker collection and measurement criteria, age scope of the subjects, sample size, statistical analysis technique (many studies have employed linear regression to display the age sequence of Aβ42, which may ignore the nonlinear trajectory), and, most importantly, the *APOE* ε4 status. As another core biomarker of amyloid plaque in the AT(N) framework (Fleisher et al. 2015; Jack Jr. et al. 2018), the Aβ42/Aβ40 ratio showed a linear decreasing pattern across all age ranges (18–93 years) in the present study, and no inflection time points or *APOE* interaction effect were found. The Aβ42/Aβ40 ratio seems to be a biomarker of aging accumulation in the cognitively normal stage.

Among *APOE* ε4+ subjects, the age‐related accelerated changes in CSF P‐tau (at ≈59 years of age) and T‐tau (at ≈57 years of age) occurred almost simultaneously and significantly later (nearly 11–13 years) than that noted for CSF Aβ42 (at ≈46 years of age). In the *APOE* ε4− group, both P‐tau and T‐tau levels gradually increased without obvious inflection points. These findings suggested that CSF P‐tau and T‐tau levels may change synchronously during the aging process, and the temporal evolution of these changes in *APOE* ɛ4 carriers may lag behind the changes in Aβ42 levels by at least a decade. However, consistent with previous studies (Li et al. 2017; Morris et al. 2010), no interaction effect of *APOE* ε4 status was found for the association between P‐tau or T‐tau levels and age in the present study. Future investigations of the effects of different *APOE* ε4 status on the associations between P‐tau and T‐tau levels and age within *APOE* ε4 subgroups determined via RCS tests or other models are necessary to assess this possible interpretation of our findings. CSF P‐tau and T‐tau levels are acknowledged as downstream biomarkers of CSF Aβ42 in AD, but their relationships in CN patients have hardly been explored (Alcolea et al. 2015; Fleisher et al. 2015). In this study, P‐tau was positively associated with Aβ42 in both the younger cohort and the older cohort, and higher P‐tau levels were more strongly associated with higher Aβ42 levels in younger subjects than in older subjects. Opposite to P‐tau, T‐tau was negatively associated with Aβ42 levels with a marginal significance (*p =* 0.05) only in the older cohort. One previous study has indicated that higher T‐tau levels were associated with lower Aβ42 levels in individuals with Aβ42 concentrations less than 550 pg/mL (Alcolea et al. 2015). As greater age corresponds to lower Aβ42 levels, our study is partly consistent with previous studies and further revealed a potential cut‐off time point for the association between Aβ42 and T‐tau levels in CN subjects.

Our temporal evolution discoveries of AT(N) framework biomarkers (Aβ42, the Aβ42/Aβ40 ratio, P‐tau, and T‐tau) (Jack Jr. et al. 2018) are similar to the findings from European and American population‐based research (Fortea et al. 2024). These findings revealed a strong gene dose effect of *APOE* ε4 status on biomarker trajectories of the AT(N) classification system, emphasizing that *APOE* homozygotes should be considered another marker of genetically determined AD and further explored. Specifically, the study assessed and compared the age at which divergence occurs and revealed that the CSF Aβ42 trajectory of *APOE* ε4 and *APOE* ε3 homozygotes clearly deviated as early as 50 years of age (both with a decreasing pattern from 50 to 90 years of age), and CSF P‐tau levels in *APOE* ε4 homozygotes started an accelerated ascent in participants in their early 50s and gradually decelerated after the age of approximately 70 years. CSF P‐tau levels in *APOE* ε3 individuals increase after 50 years of age and gradually accelerate throughout the lifespan. In this study, age‐related changes in P‐tau and Aβ42 levels in individuals between 50 and 90 years of age followed similar trajectories, with subtle deviations in inflection time points. Individuals aged less than 50 years and CSF T‐tau analyses were not included in the study (Fortea et al. 2024). In our study, we found unexpected positive directions of change in CSF Aβ42 levels at the ages noted before inflection points, which should be further validated in a specific young cohort. Future studies separating *APOE* ε4 carriers into heterozygote and homozygote categories are needed to elucidate the full scope of the effect of *APOE* ε4 status on biomarker trajectories in Asian populations.

The study indicated that CSF NSE, H‐FABP, VILIP‐1, and α‐synuclein were positively correlated with CSF Aβ42 levels in the younger group instead of the older group. It added more detailed information on the relationships between Aβ42 and these biomarkers to previous studies (Alcolea et al. 2015; Tarawneh et al. 2011). We assume that the differences between younger and older groups may be attributed to the possibility that older individuals are confounded with many other pathologies that may affect the levels of these biomarkers (Mehta and Schneider 2021). These biomarkers are not specific for AD but are nonspecific indices of damage derived from many other etiologies. Surprisingly, the correlations between these biomarkers and Aβ42 in our CN subjects were completely different from those in AD subjects (Hoglund et al. 2017; Olsson et al. 2016; Toledo et al. 2013), suggesting a negative association. Contrary to the relationship of Aβ42 with other biomarkers, Aβ42/Aβ40 ratio shows a negative correlation with other biomarkers, which is consistent with the relationship observed in AD patients. We speculate that during the aging process, the pathophysiological connection of Aβ42/Aβ40 ratio with other biomarkers may closely resemble that observed in AD patients, and the mechanisms by which amyloid‐β affects these biomarkers‐related neuronal injury/degeneration, neuroinflammation, or α‐synuclein may be different between AD and CN individuals. More fundamental research is needed to explore the underlying mechanisms involved.

This study has several limitations. Firstly, the cross‐sectional nature of our study may not fully capture the longitudinal alterations in biomarkers over time. The reliance on cross‐sectional data limits our ability to infer causality or assess temporal changes in biomarker levels. Additionally, despite adjusting for multiple confounders, residual confounding remains a possibility. Future research should prioritize longitudinal cohort studies to provide a more comprehensive understanding of the temporal dynamics of these biomarkers. Secondly, the limited sample size affects the reliability and generalizability of our findings. Thirdly, the absence of neuroimaging for all participants to rule out conditions such as stroke and small vessel diseases represents another limitation of the study. Future studies should aim to address these issues by incorporating larger sample sizes, longitudinal cohorts, and more refined participant stratification to validate our current findings.

## 5 Conclusion

The evolution of AD‐related biomarkers in CSF varies throughout the adult lifespan, with the *APOE* ε4 allele modifying the temporal changes in CSF Aβ42 levels, as well as potentially influencing P‐tau and T‐tau levels.

## Author Contributions

Yan‐Jiang Wang, Jun Wang, and Zhen Wang designed this study. Wei‐Wei Li, Jun Wang, and Yan‐Jiang Wang drafted the manuscript. Wei‐Wei Li and Dong‐Yu Fan performed the experiments. Wei‐Wei Li and Lei‐Kai Wang analyzed the data and presented the results. Qi Sun, Bing‐Qiang Huang, Ying‐Ying Shen, Cheng‐Rong Tan, and Gui‐Hua Zeng collected the CSF samples and clinical information. Zhong‐Yuan Yu, Ding‐Yuan Tian, Fan Zeng, and Jin Fan had critical reading of the manuscript.

## Conflicts of Interest

The authors declare no conflicts of interest.

## Supporting information


Data S1.

## Data Availability

The data evaluating the conclusions in the article are present in the article and the Supporting Information. Additional data supporting the research findings are available on reasonable request from the corresponding author.
