# Supplementary material for: Temporal Changes in Alzheimer's Disease‐Related Biomarkers in the CSF of Cognitively Normal Subjects at Different Ages: The Chongqing Ageing and Dementia Study
Source: Aging Cell. 2025 Mar 9;24(6):e70036. doi: 10.1111/acel.70036 (PMC12151905; doi:10.1111/acel.70036)
Supplement: Supplementary file 1 — Data S1. [file ACEL-24-e70036-s001.docx]

**Supplementary Information**

**Title:** **Temporal changes in Alzheimer’s disease-related biomarkers in the CSF of cognitively normal subjects at different ages: the Chongqing Ageing and Dementia Study**

Wei-Wei Li ^1,2,#^, Dong-Yu Fan ^1,3,#^, Qi Sun ^4,#^, Lei-Kai Wang ^5,#^, Bing-Qiang Huang ^4^, Zhong-Yuan Yu ^1,6^, Ding-Yuan Tian ^1^, Ying-Ying Shen ^1,6^, Cheng-Rong Tan ^1,6^, Gui-Hua Zeng ^1,6^, Fan Zeng ^1,6^, Jin Fan ^2^, Zhen Wang ^4,*^, Yan-Jiang Wang^1,6,*^, Jun Wang ^1,6,*^

^1^ Department of Neurology and Centre for Clinical Neuroscience, Daping Hospital, Third Military Medical University, Chongqing, China.

^2^ Department of Neurology, The General Hospital of Western Theater Command, Chengdu, China.

^3^ Department of Plateau Diseases, Shigatse Branch, Xinqiao Hospital, Third Military Medical University, Shigatse, China.

^4^ Department of Anesthesiology, Daping Hospital, Third Military Medical University, Chongqing, China.

^5^ Department of Neurosurgery, The General Hospital of Western Theater Command, Chengdu, China.

^6^ Chongqing Key Laboratory of Aging and Brain Diseases, Chongqing, China.

^#^ These authors equally contributed to this study.

^*^ Correspondence to: Yan-Jiang Wang, Email: [yanjiang_wang@tmmu.edu.cn](mailto:yanjiang_wang@tmmu.edu.cn); Zhen Wang, Email: dpicuwz@tmmu.edu.cn; or Jun Wang, Email: [qywangjun@163.com](mailto:qywangjun@163.com).

**Supplementary Tables**

**Table S1 ELISA kits used in this study.**

| **Biomarkers** | **ELISA kits** | **Brand** | **Catalog number** | **Sample Dilution** |
| --- | --- | --- | --- | --- |
| Aβ42 | INNOTEST® β-AMYLOID(1-42) | Fujirebio | 81584 | Without dilution |
| Aβ40 | INNOTEST® β-AMYLOID(1-40) | Fujirebio | 81586 | 100 fold |
| P-tau | INNOTEST® PHOSPHO-TAU (181P) | Fujirebio | 81582 | Without dilution |
| T-tau | INNOTEST® hTAU Ag | Fujirebio | 81580 | Without dilution |
| NSE | Human Enolase 2/Neuron-specific Enolase Quantikine ELISA Kit | R&D | DENL20 | 2 fold |
| H-FABP | Human H-FABP ELISA kit | Hycult | HK402 | Without dilution |
| VILIP-1 | Human VILIP-1 ELISA kit | Biovender | RD191119200R | Without dilution |
| YKL-40 | Human CHI3L1 ELISA Kit | Raybiotech | ELH-CHI3L1-1 | 1000 fold |
| TREM2 | Human TREM2 ELISA Kit | Arigobio | ARG81633 | 10 fold |
| α-synuclein | Human α-Synuclein (Colorimetric) ELISA Kit | Biolegend  (covance) | 844101 | 10 fold |

The NFL levels were measured with an ultrasensitive single-molecule array (SIMOA) on a Simoa HD-1 analyser (Quanterix, Lexington, Massachusetts).

**Table S2 Data** **availability for all variables and the missing data patterns**

| **Biomarkers or Demographic data** | ***APOE* ε4+ group (n)** | ***APOE* ε4-group (n)** | **Total (n)** | **Missing number (n)** | **Missing ratio (%)** |
| --- | --- | --- | --- | --- | --- |
| Aβ42* | 100 | 397 | 522 | 27 | 4.9 |
| Aβ40* | 98 | 395 | 520 | 29 | 5.3 |
| P-tau* | 100 | 397 | 522 | 27 | 4.9 |
| T-tau* | 98 | 401 | 525 | 24 | 4.4 |
| NFL* | 100 | 402 | 529 | 20 | 3.6 |
| NSE* | 87 | 337 | 447 | 102 | 18.6 |
| H-FABP* | 92 | 341 | 452 | 97 | 17.7 |
| VILIP-1* | 84 | 325 | 431 | 118 | 21.5 |
| YKL-40* | 87 | 335 | 447 | 102 | 18.6 |
| TREM2* | 88 | 333 | 440 | 109 | 19.9 |
| α-synuclein* | 86 | 335 | 438 | 111 | 20.2 |
| age | 104 | 415 | 549 | 0 | 0 |
| sex | 104 | 415 | 549 | 0 | 0 |
| education# | 59 | 233 | 303 | 246 | 44.8 |
| **smoke**# | 104 | 413 | 547 | 2 | 0.4 |
| **alcohol**# | 104 | 413 | 547 | 2 | 0.4 |
| **hyperlipemia**# | 104 | 413 | 547 | 2 | 0.4 |
| **hypertension**# | 104 | 413 | 547 | 2 | 0.4 |
| **diabetes**# | 104 | 413 | 547 | 2 | 0.4 |
| **CHD**# | 104 | 413 | 547 | 2 | 0.4 |

The precise data of accessible data in the total cohort and *APOE* ɛ4 subgroup.

*Missing data points due to inadequate CSF volume for assessment or specimen contamination during the assays.

# Missing data points due to loss of collection.

*APOE* genotyping was performed for 519 subjects (30 missing).

Abbreviations: *APOE*, apolipoprotein E; CHD, coronary heart disease history.

**Table S3 RCS analyses of biomarkers and age in total and *APOE* ε4-stratified cohorts**

| Biomarkers | **All** | | | |  | ***APOE* ε4+ group (n=104)** | | | |  | ***APOE* ε4- group (n=415)** | | | | *p* for interaction  (*APOE* ε4*age for RCS) |
| --- | --- | --- | --- | --- | --- | --- | --- | --- | --- | --- | --- | --- | --- | --- | --- |
|  | *p*-overall | *p*-nonlinear | knots | inflection point |  | *p-*overall | *p*-nonlinear | knots | inflection point |  | *p-*overall | *p-*nonlinear | knots | inflection point |  |
| Aβ42 | <0.0001 | **<0.0001** | 5 | 67.48y |  | <0.0001 | **0.002** | 3 | 45.69y |  | 0.0004 | **0.001** | 5 | 68.02y | **0.003** |
| Aβ40 | <0.0001 | 0.06 |  |  |  |  |  |  |  |  |  |  |  |  |  |
| Aβ42/Aβ40 ratio | 0.015 | 0.86 |  |  |  |  |  |  |  |  |  |  |  |  |  |
| P-tau | <0.0001 | **0.008** | 3 | 54.65y |  | <0.0001 | **0.02** | 6 | 59.24y |  | <0.0001 | 0.06 | 3 | NA | 0.50 |
| T-tau | <0.0001 | **0.003** | 3 | 54.83y |  | 0.0001 | **0.04** | 6 | 56.94y |  | <0.0001 | **0.01** | 3 | NA | 0.24 |
| NFL | <0.0001 | 0.96 |  |  |  |  |  |  |  |  |  |  |  |  |  |
| NSE | <0.0001 | 0.08 |  |  |  |  |  |  |  |  |  |  |  |  |  |
| H-FABP | <0.0001 | 0.20 |  |  |  |  |  |  |  |  |  |  |  |  |  |
| VILIP-1 | 0.0002 | 0.35 |  |  |  |  |  |  |  |  |  |  |  |  |  |
| YKL-40 | <0.0001 | 0.45 |  |  |  |  |  |  |  |  |  |  |  |  |  |
| TREM2 | 0.001 | 0.72 |  |  |  |  |  |  |  |  |  |  |  |  |  |
| α-synuclein | 0.25 | 0.67 |  |  |  |  |  |  |  |  |  |  |  |  |  |

Total and *APOE* ɛ4-stratified analyses of age-related changes in all biomarkers were modelled with RCSs adjusted by covariates, and the biomarkers with significant nonlinear associations (*p*-nonlinear<0.05) were displayed with detailed information including knots, inflection point and the interaction effect of *APOE* ε4*age. The selection of the knots in the RCS curve were based on the principle of minimum AIC. The bolded p-nonlinear indicates significant result.

Abbreviations: RCS, restricted cubic splines; *APOE*, apolipoprotein E; AIC, Akaike Information Criterion, y, years of age.

**Table S4 Interaction analyses of *APOE*** **ε4 status and age in linear regression**

| CSF biomarkers | log-transformed | **age** | | | |  | **age**APOE* ε4 status** | | | |
| --- | --- | --- | --- | --- | --- | --- | --- | --- | --- | --- |
|  |  | β | unstandardized coefficients (95%CI) | SE | *p* value |  | β | unstandardized coefficients (95%CI) | SE | *p* for interaction |
| Aβ40 | No | 0.24 | 54.70(31.32~78.08) | 11.90 | **<0.0001** |  | -0.12 | -21.88(-65.65~ 21.88) | 22.27 | 0.33 |
| Aβ42/Aβ40 ratio | No | -0.16 | -0.0008-0.001~ -0.0002) | 0.0003 | **0.004** |  | 0.13 | 0.00005(-0.0009~ 0.001) | 0.0005 | 0.92 |
| NFL | yes | 0.51 | 0.02(0.02~0.03) | 0.002 | **<0.0001** |  | 0.06 | 0.002(-0.005~0.009) | 0.004 | 0.58 |
| NSE | no | 0.33 | 56.83(37.75~75.92) | 9.71 | **<0.0001** |  | -0.06 | -8.03(-43.21~ 27.14) | 17.90 | 0.65 |
| H-FABP | no | 0.36 | 3.17(2.19~4.15) | 0.50 | **<0.0001** |  | -0.13 | -0.88(-2.65~0.88) | 0.90 | 0.33 |
| VILIP-1 | no | 0.24 | 0.89(0.45~1.32) | 0.22 | **<0.0001** |  | -0.09 | -0.28(-1.05~0.50) | 0.39 | 0.48 |
| YKL-40 | no | 0.46 | 4.98(3.88~6.08) | 0.56 | **<0.0001** |  | 0.13 | 1.08(-0.95~3.11) | 1.03 | 0.29 |
| TREM2 | yes | 0.29 | 0.007(0.004~0.01) | 0.001 | **<0.0001** |  | -0.17 | -0.003(-0.008~ 0.002) | 0.003 | 0.20 |
| α-synuclein | yes | 0.15 | 0.007(0.001~0.01) | 0.003 | **0.01** |  | -0.27 | -0.01(-0.02~-0.00006) | 0.005 | 0.05 |

Linear regression analyses, adjusted for covariates, were conducted along with interaction analyses involving *APOE* ε4 status and age. Biomarkers were log-transformed when necessary to meet the “LINE” standards of residual, including linearity, independence, normality and homoscedasticity. The bolded p-value indicates significant result.

Abbreviations: CSF, cerebrospinal fluid; *APOE*, apolipoprotein E; β, standardized coefficient; 95%CI, 95% confidence interval; SE, standard error.

**Table S5 Linear regression analyses of biomarkers and age in *APOE* ε4-stratified cohorts**

| CSF biomarkers | log-transformed | ***APOE* ε4+** | | | |  | ***APOE* ε4-** | | | |
| --- | --- | --- | --- | --- | --- | --- | --- | --- | --- | --- |
|  |  | β | unstandardized coefficients (95%CI) | SE | *p* value |  | β | unstandardized coefficients (95%CI) | SE | *p* value |
| Aβ40 | No | 0.12 | 24.55(-19.28~ 68.38) | 22.08 | **0.27** |  | 0.25 | 56.96(33.10~80.82) | 12.14 | **<0.0001** |
| Aβ42/Aβ40 ratio | No | -0.22 | -0.0008(-0.002~0.000008) | 0.0004 | **0.05** |  | -0.15 | -0.0007(-0.001~ -0.0002) | 0.0003 | **0.008** |
| NFL | yes | 0.64 | 0.02(0.02~0.03) | 0.003 | **<0.0001** |  | 0.49 | 0.02(0.02~0.03) | 0.002 | **<0.0001** |
| NSE | no | 0.30 | 46.45(11.66~ 81.24) | 17.50 | **0.009** |  | 0.33 | 58.21(38.90~77.53) | 9.82 | **<0.0001** |
| H-FABP | no | 0.40 | 2.69(1.31~4.07) | 0.70 | **0.0002** |  | 0.32 | 3.04(2.001~4.08) | 0.53 | **<0.0001** |
| VILIP-1 | no | 0.23 | 0.84(-0.04~1.72) | 0.44 | 0.06 |  | 0.23 | 0.84(0.41~1.26) | 0.22 | **0.0001** |
| YKL-40 | no | 0.72 | 6.62(4.99~8.26) | 0.82 | **<0.0001** |  | 0.43 | 4.89(3.74~6.05) | 0.59 | **<0.0001** |
| TREM2 | yes | 0.19 | 0.004(-0.001~ 0.009) | 0.003 | 0.12 |  | 0.28 | 0.007(0.004~0.01) | 0.001 | **<0.0001** |
| α-synuclein | yes | -0.04 | -0.002(-0.01~ 0.009) | 0.005 | 0.73 |  | 0.14 | 0.007(0.001~0.01) | 0.003 | **0.02** |

Biomarkers were log-transformed when necessary to meet the “LINE” standards of residual, including linearity, independence, normality and homoscedasticity. The bolded p-value indicates significant result.

Abbreviations: CSF, cerebrospinal fluid; *APOE*, apolipoprotein E; β, standardized coefficient; 95%CI, 95% confidence interval; SE, standard error.

**Table S6** **Correlations across each pairing of biomarkers**

| Biomarker correlations | Aβ42/Aβ40 ratio | P-tau | T-tau | NFL | NSE | HFABP | VILIP-1 | YKL-40 | TREM2 | α-synuclein |
| --- | --- | --- | --- | --- | --- | --- | --- | --- | --- | --- |
| Aβ42 | 0.19(<0.001) | 0.31(<0.001) | 0.003(0.95) | 0.09(0.046) | 0.15(0.002) | 0.11(0.02) | 0.33(<0.001) | 0.01(0.81) | 0.07(0.15) | 0.26(<0.001) |
| Aβ42/Aβ40 ratio |  | **-0.36(<0.001)** | **-0.33(<0.001)** | **-0.23(<0.001)** | **-0.34(<0.001)** | **-0.25(<0.001)** | **-**  **0.16(0.001)** | **-0.19(<0.001)** | **-**  **0.11(0.02)** | -  0.02(0.63) |
| P-tau |  |  | 0.74(<0.001) | 0.31(<0.001) | 0.55(<0.001) | 0.52(<0.001) | 0.59(<0.001) | 0.42(<0.001) | 0.26(<0.001) | 0.21(<0.001) |
| T-tau |  |  |  | 0.36(<0.001) | 0.53(<0.001) | 0.58(<0.001) | 0.46(<0.001) | 0.44(<0.001) | 0.27(<0.001) | 0.17(<0.001) |
| NFL |  |  |  |  | 0.32(<0.001) | 0.46(<0.001) | 0.28(<0.001) | 0.40(<0.001) | 0.29(<0.001) | 0.07(0.16) |
| NSE |  |  |  |  |  | 0.48(<0.001) | 0.41(<0.001) | 0.44(<0.001) | 0.20(<0.001) | 0.18(<0.001) |
| HFABP |  |  |  |  |  |  | 0.46(<0.001) | 0.46(<0.001) | 0.36(<0.001) | 0.19(<0.001) |
| VILIP-1 |  |  |  |  |  |  |  | 0.28(<0.001) | 0.19(<0.001) | 0.40(<0.001) |
| YKL-40 |  |  |  |  |  |  |  |  | 0.25(<0.001) | 0.07(0.22） |
| TREM2 |  |  |  |  |  |  |  |  |  | 0.17(<0.001) |

Spearman correlation analyses across each pairing of biomarkers displayed with correlation matrix table. Data were shown as correlation coefficients (*p* value). The bolded texts indicate negative associations; The grey-filled text indicate no associations; The others indicate positive associations.

**Table S7 Interaction analyses of sex and age in linear regression**

| CSF biomarkers | log-transformed | **age** | | | |  | **age*sex** | | | |
| --- | --- | --- | --- | --- | --- | --- | --- | --- | --- | --- |
|  |  | β | unstandardized coefficients (95%CI) | SE | p value |  | β | unstandardized coefficients (95%CI) | SE | *p* for interaction |
| Aβ40 | No | 0.22 | 49.39(28.56~70.23) | 10.60 | **<0.0001** |  | 0.16 | 24.39(-21.33~78.35) | 26.92 | 0.37 |
| Aβ42/Aβ40 ratio | No | -0.16 | -0.0007(-0.001~ -0.0003) | 0.0002 | **0.001** |  | -0.05 | -0.0006(-0.001~ 0.0008) | 0.0006 | 0.33 |
| NFL | yes | 0.52 | 0.02(0.02~0.03) | 0.002 | **<0.0001** |  | 0.36 | 0.01(0.002~0.02) | 0.004 | **0.02** |
| NSE | no | 0.32 | 54.81(37.93~71.68) | 8.59 | **<0.0001** |  | -0.05 | -54.08(-99.06~-9.10) | 22.89 | **0.02** |
| H-FABP | no | 0.33 | 2.94(2.07~3.80) | 0.44 | **<0.0001** |  | -0.16 | -0.95(-3.25~1.36) | 1.17 | 0.42 |
| VILIP-1 | no | 0.22 | 0.81(0.43~1.20) | 0.19 | **<0.0001** |  | 0.08 | 0.20(-0.35~1.63) | 0.53 | 0.71 |
| YKL-40 | no | 0.49 | 5.25(4.28~6.22) | 0.49 | **<0.0001** |  | -0.06 | -0.46(-3.05~2.13) | 1.32 | 0.73 |
| TREM2 | yes | 0.25 | 0.006(0.004~0.009) | 0.001 | **<0.0001** |  | -0.20 | -0.003(-0.01~0.003) | 0.003 | 0.33 |
| α-synuclein | yes | 0.09 | 0.004(-0.0006~0.009) | 0.003 | 0.08 |  | -0.18 | -0.006(-0.02~0.008) | 0.007 | 0.41 |

Linear regression analyses, adjusted for covariates, were conducted along with interaction analyses involving sex and age. Biomarkers were log-transformed when necessary to meet the “LINE” standards of residual, including linearity, independence, normality and homoscedasticity. The bolded p-value indicates significant result.

Abbreviations: CSF, cerebrospinal fluid; β, standardized coefficient; 95%CI, 95% confidence interval; SE, standard error.

**Figure S1** **Sex differences in the associations between core biomarkers and age.**

**
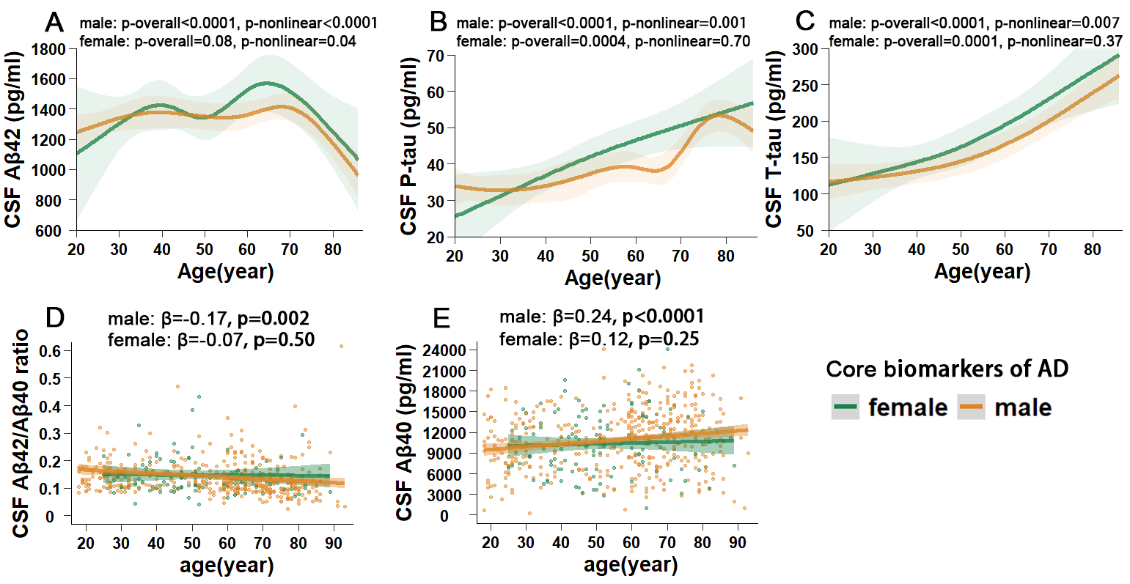
**

Sex-stratified analyses of age-related changes in CSF Aβ42 (A), P-tau (B), and T-tau (C) were modelled with RCSs adjusted by covariates. Here, p-overall<0.05 referred to a significant association (linear or nonlinear), and p-nonlinear<0.05 referred to a nonlinear association between biomarkers and age. Aβ42/Aβ40 ratio (D), and Aβ40 (E) were modelled with linear regressions adjusted by covariates. Shading around the curve or regression line represents standard errors. RCS, restricted cubic splines; β, standardized coefficient.

**Figure S2 Sex differences in** **the linear associations between other biomarkers and age.**

**
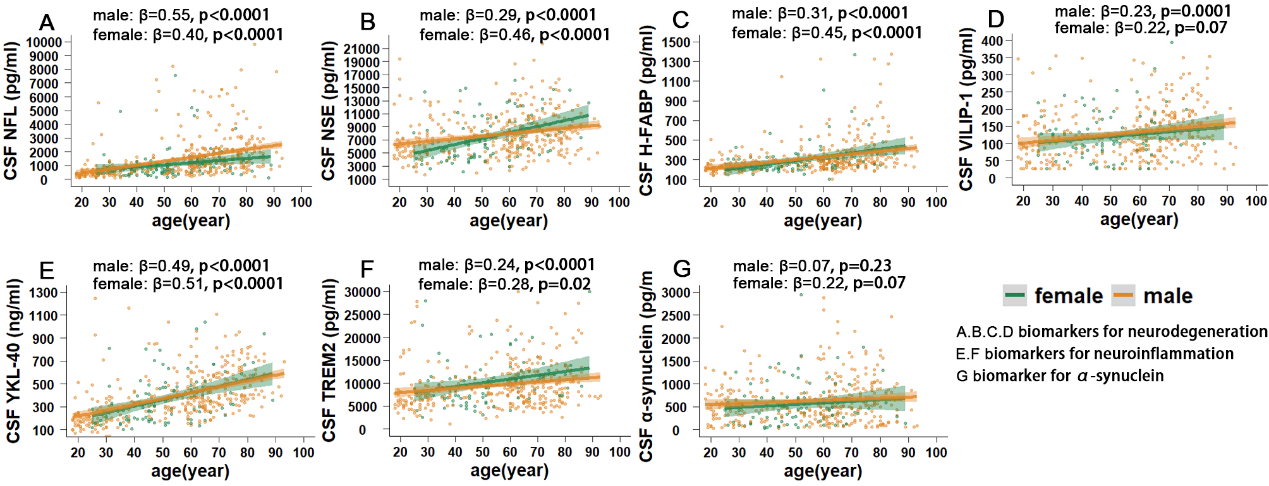
**

Sex-stratified analyses of the age-related changes in the CSF NFL (A), NSE (B), H-FABP (C), VILIP-1 (D), YKL-40 (E), TREM2 (F) and α-synuclein (G) were modelled with linear regressions adjusted by covariates. Shading around the regression line represents standard errors. β, standardized coefficient.
